# A fibrin/hyaluronic acid hydrogel for the delivery of mesenchymal stem cells and potential for articular cartilage repair

**DOI:** 10.1186/1754-1611-8-10

**Published:** 2014-05-01

**Authors:** Timothy N Snyder, Krishna Madhavan, Miranda Intrator, Ryan C Dregalla, Daewon Park

**Affiliations:** 1 Bioengineering Department, University of Colorado, Anschutz Medical Campus, Mail Stop 8607, 12700 East 19th Avenue, Aurora, CO 80045, USA; 2 Regenerative Sciences, 403 Summit Blvd, Suite 201, Broomfield, CO 80021, USA

**Keywords:** Osteoarthritis, Fibrin, Hyaluronic acid, Mesenchymal stem cell, Hydrogel, Cartilage, Stem cell delivery, Regenerative medicine, Tissue engineering

## Abstract

**Background:**

Osteoarthritis (OA) is a degenerative joint disease affecting approximately 27 million Americans, and even more worldwide. OA is characterized by degeneration of subchondral bone and articular cartilage. In this study, a chondrogenic fibrin/hyaluronic acid (HA)-based hydrogel seeded with bone marrow-derived mesenchymal stem cells (BMSCs) was investigated as a method of regenerating these tissues for OA therapy. This chondrogenic hydrogel system can be delivered in a minimally invasive manner through a small gauge needle, forming a three-dimensional (3D) network structure *in situ*. However, an ongoing problem with fibrin/HA-based biomaterials is poor mechanical strength. This was addressed by modifying HA with methacrylic anhydride (MA) (HA-MA), which reinforces the fibrin gel, thereby improving mechanical properties. In this study, a range of fibrinogen (the fibrin precursor) and HA-MA concentrations were explored to determine optimal conditions for increased mechanical strength, BMSC proliferation, and chondrogenesis potential *in vitro*.

**Results:**

Increased mechanical strength was achieved by HA-MA reinforcement within fibrin hydrogels, and was directly correlated with increasing HA-MA concentration. Live/dead staining and metabolic assays confirmed that the crosslinked fibrin/HA-MA hydrogels provided a suitable 3D environment for BMSC proliferation. Quantitative polymerase chain reaction (qPCR) of BMSCs incubated in the fibrin/HA-MA hydrogel confirmed decreased expression of collagen type 1 alpha 1 mRNA with an increase in Sox9 mRNA expression especially in the presence of a platelet lysate, suggesting early chondrogenesis.

**Conclusion:**

Fibrin/HA-MA hydrogel may be a suitable delivery method for BMSCs, inducing BMSC differentiation into chondrocytes and potentially aiding in articular cartilage repair for OA therapy.

## Introduction

Over 30 million Americans suffer from arthritis and other rheumatic (affecting joints and connective tissue) conditions, and by 2030 nearly 25% of the American population is expected to be affected by such conditions [1]. Osteoarthritis (OA) represents the majority of these cases, and is characterized by loss of articular cartilage and subchondral bone. This condition causes severe pain, and is the leading cause of chronic disability in the U.S. [1-3]. Articular cartilage damage in particular is difficult to treat due to the lack of nerves and vasculature in the cartilage [4]. Some progress has been made in the past two decades, and regeneration of injured or degenerated cartilage has been achieved using surgical techniques such as debridement [5], microfracture [6-8], mosaicplasty [9], perichondral grafting [10,11] and periosteal grafting [12,13]. More recently, research has focused on the use of synthetic implants [14,15] for cartilage regeneration. However, about 20% of synthetic implants are known to fail approximately a decade after implantation [16]. Furthermore, this method introduces other surgery-associated risks due to the need for surgical implantation [17]. The delivery of autologous chondrocytes [18-21] or mesenchymal stem cells (MSCs) [22-25] for cartilage regeneration has shown some promising results. MSCs in particular have received much attention in cartilage regeneration, as they are multipotent cells capable of differentiating into cartilage, bone, muscle, fat and marrow stroma in response to appropriate signaling pathways [26]. However, because autologous chondrocytes and MSCs are highly environment-sensitive, their use has been severely hampered by the lack of suitable delivery methods [27]. For example, unfavorable conditions of the delivery matrix have led to autologous chondrocyte differentiation into fibroblast-like cells, which form fibrous tissues that can prevent healing [28].

Hyaluronic acid (HA) scaffolds have been well established as a biomaterial [29] for MSC delivery. HA hydrogels have been used to induce MSC osteogenesis [30,31], adipogenesis [32] and keratinogenesis [32]*in vitro*. Chung et al reported that delivery of MSCs in HA hydrogels promoted chondrogenic gene expression [33]. HA is known to directly interact with the fibrin precursor fibrinogen through reversible complex ionic interactions [34], yet it provides a sufficiently non-adhesive surface that allows cells to move more freely [35]. Fibrin scaffolds have also been investigated for MSC delivery both *in vitro *[23,24] and *in vivo *[24,36-38]. A recent study concluded that a system comprising bone marrow-derived MSCs (BMSCs) embedded within fibrin gels with TGFβ3 was able to stimulate appropriate differentiation and articular cartilage regeneration both *in vitro* and *in vivo *[23,39].

In general, these chondrogenic hydrogels should be biocompatible, possess sufficient mechanical strength, allow good adhesion of MSCs, and permit diffusion of nutrients and differentiation factors to induce chondrogenesis and hence cartilage repair [40]. Pure unmodified fibrin hydrogels possess excellent biocompatibility since they contain native arginine-glycine-aspartic acid (RGD) sites for MSC attachment [41]; however, without reinforcements or crosslinking, they possess low mechanical strength [42-45]. Although HA hydrogels possess better mechanical strength, they lack adhesive motifs [31] and thus deter cell proliferation at higher concentrations (>1 mg/mL) [30,46].

In this study, to exploit the advantages of both fibrin and HA, we fabricated a fibrin gel reinforced by crosslinked hyaluronic acid methacrylate (HA-MA). This fibrin/HA-MA hydrogel was examined to determine its *in vitro* mechanical and biological properties, and chondrogenesis was examined by BMSCs in the hydrogel. We hypothesized that the combination of fibrin and HA-MA would promote cell attachment and proliferation, and subsequently induce chondrogenesis, and thus cartilage regeneration and repair.

## Results and discussion

### BMSC phenotype verification

**Figure 1:**
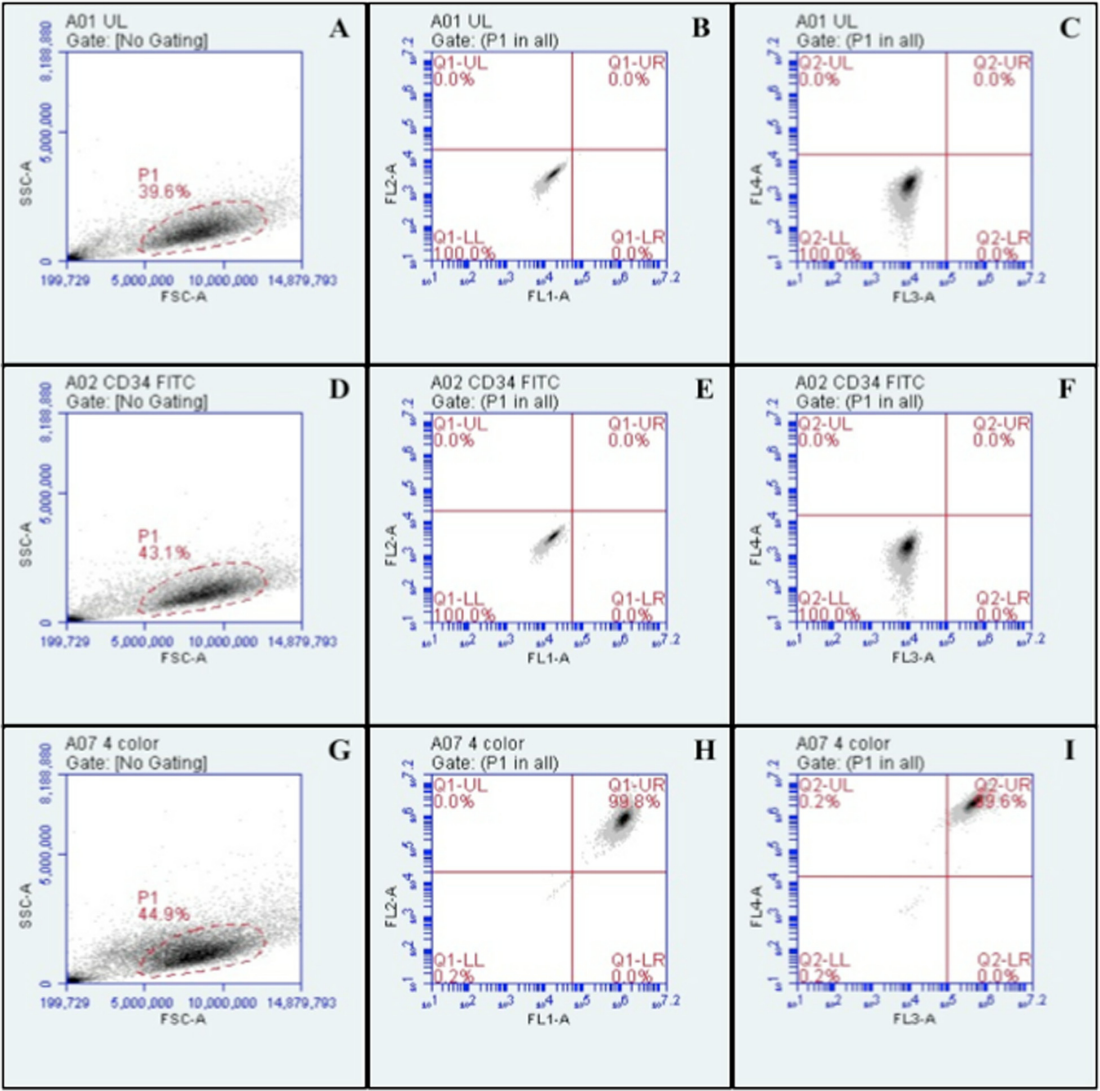
**Flow cytometry verifies BMSC phenotype via positive and negative surface markers.** Unlabeled BMSCs: **(A)** FSC vs. SSC and **(B-C)** Absence of fluorescence detected through any channels. BMSCs treated with CD34-FITC indicate no CD34 expression: **(D)** FSC vs. SSC, **(E)** all cells were FITC-negative as detected through channels FL1 and FL2 (100.0% in Q1-LL) and **(F)** through channels FL3 and FL4 (100.0% in Q2-LL). BMSCs treated with CD105-PE, CD73-PerCP, CD90-APC, CD44-FITC indicate expression of all four surface proteins: **(G)** FSC vs. SSC, **(H)** 99.8% of cells (Q1-UR) were PE, PerCP, APC and FITC-positive as detected through FL1 and FL2 and **(I)** 99.6% of cells (Q2-UR) were detected positive through FL3 and FL4.

Phenotypical surface protein expression of BMSCs was confirmed by flow cytometry (Figure 1) after incubating BMSCs with the antibodies unlabeled with fluorescent dyes (Figure 1A, B and C), anti-CD34 antibody (Figure 1D, E and F) and fluorescent-labeled antibodies, CD105-PE, CD73-PerCP, CD-90APC, CD44-FITC & CD34-FITC (Figure 1G, H and I). The first graph of each case (Figure 1A, D and G) is the side scatter channel (SSC) vs. forward scatter channel (FSC). SSC is a measure of cell granularity, while FSC represents overall cell size. The next two graphs of each case represent the fluorescent intensity through channels FL1 (fluorescein isothiocyanate, FITC) and FL2 (phycoerythrin, PE) (Figure 1B, E and H), and FL3 (peridinin chlorophyll protein complex, PerCP) and FL4 (allophycocyanin, APC) (Figure 1C, F and I). Based on the fluorescent tag of the antibodies, positive markers display a shift in their respective fluorescent channel. In the four-color graphs (Figure 1G, H and I), there is a shift to the upper right quadrant of both fluorescence graphs, which signifies a positive result for all four markers. All markers were run individually with positive results. The unlabeled cell population was used to determine the placement of the quadrants. Thus, the BMSCs were verified to possess the typical phenotype necessary for them to undergo chondrogenesis.

### HA-MA characterization

**Figure 2:**
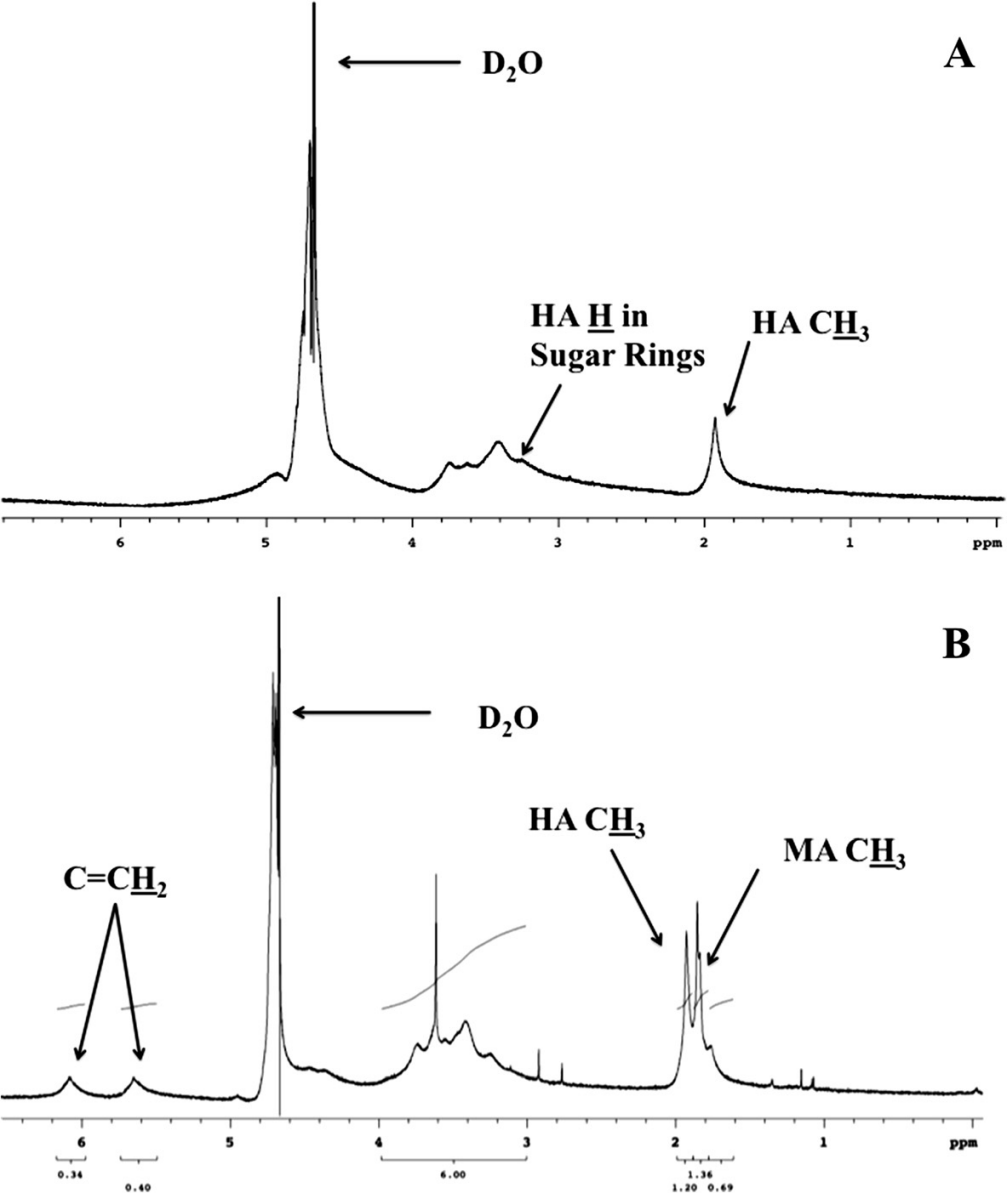
^**1**^**H NMR spectra of HA (A) and HA-MA (B) with proton peaks in the methyl groups as well as the sugar rings labeled. **^1^H for relevant peaks in each figure have been underlined.

The successful conjugation of MA to HA was confirmed by proton nuclear magnetic resonance (^1^H NMR) spectra (Figure 2). The NMR spectrum of native HA (Figure 2A) confirmed the presence of HA methyl protons at 1.9 ppm [29]. After conversion, the protons in methacrylate vinyl groups appeared at 5.6 ppm and 6.1 ppm, and the MA methyl protons were confirmed at 1.8 ppm (Figure 2B). Degree of methacrylation was determined, as described previously [47], by the ratio of the integral of the HA methyl proton peak (at ~1.9 ppm) to that of the protons of MA (at ~5.6 ppm and ~6 ppm). The degree of methacrylation was calculated as 95 ± 13%, which represents almost one methacrylate group per disaccharide unit of HA.

### BMSC viability & proliferation

**Figure 3:**
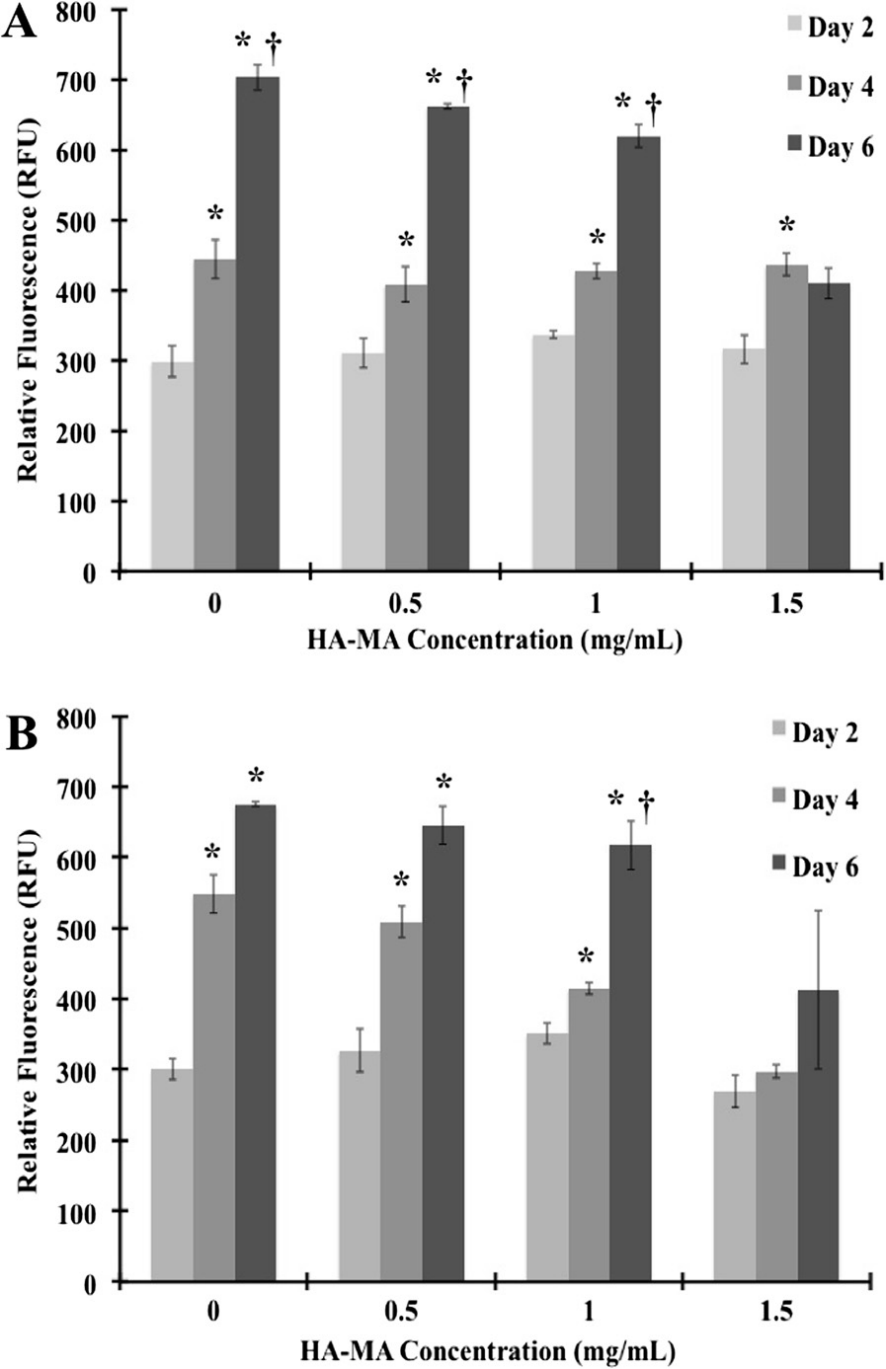
**BMSC metabolic activity in fibrin/HA-MA hydrogels with varying HA-MA concentrations. (A)** 4 mg/mL and **(B)** 6 mg/mL fibrinogen. * – Statistically significant difference between successive days for a given HA-MA concentration (p < 0.05). † – Statistically significant difference between successive HA-MA concentrations for a given day (p < 0.05).

**Figure 4:**
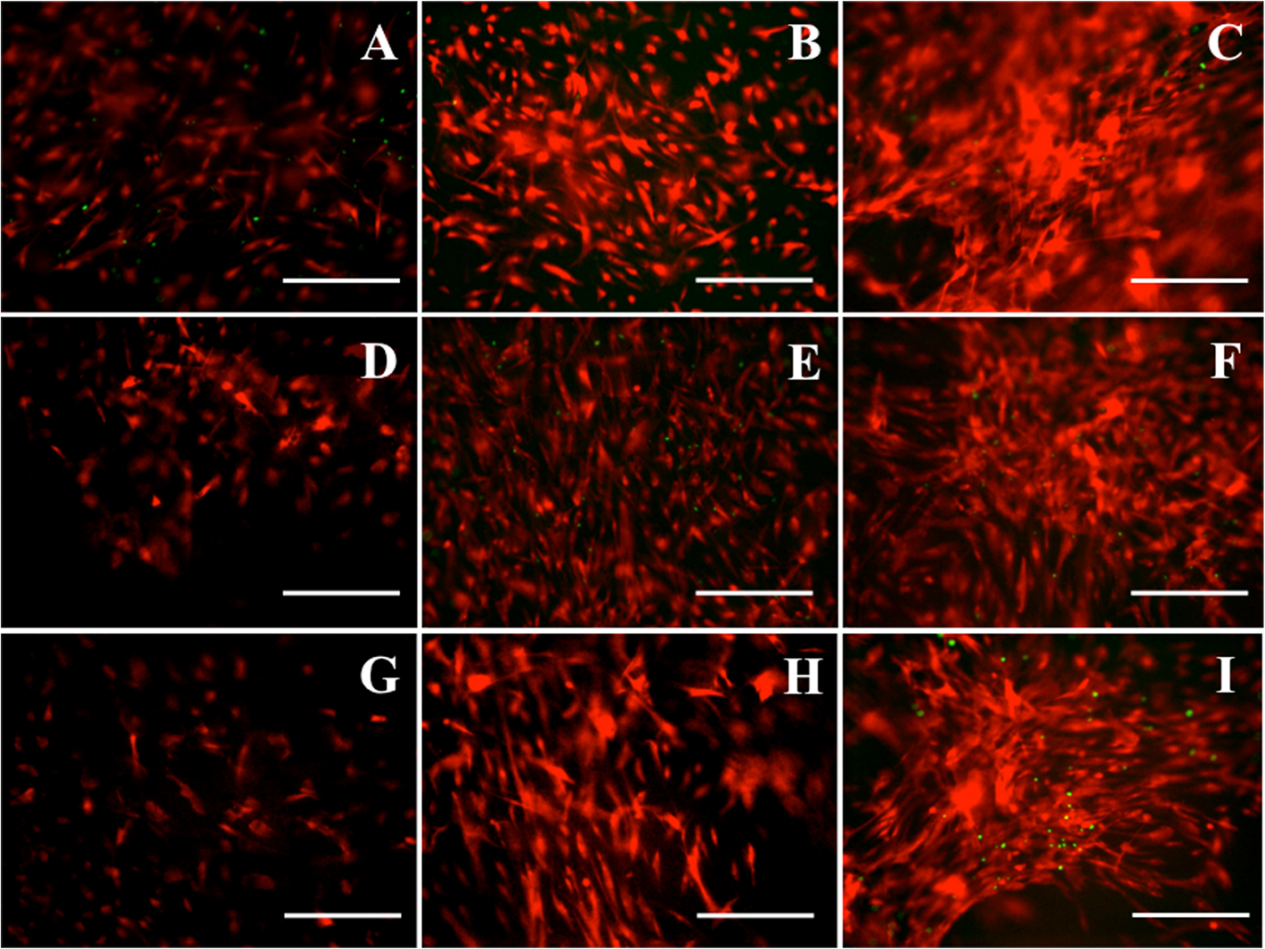
**Representative images of live/dead staining of BMSCs in fibrin/HA-MA hydrogels.** Live cells were stained with Calcein-AM (Red) and dead cells with EthD-1 (Green). Formulation with 4 mg/mL fibrinogen with 0 mg/mL HA-MA at **(A)** day 2, **(B)** day 4, and **(C)** day 6. Formulation with 4 mg/mL fibrinogen with 1 mg/mL HA-MA at **(D)** day 2, **(E)** day 4, and **(F)** day 6. Formulation with 6 mg/mL fibrinogen with 1 mg/mL HA-MA at **(G)** day 2, **(H)** day 4, and **(I)** day 6. Scale bar: 100 μm.

BMSC activity was examined using a metabolic assay after 2, 4, and 6-day culture in different fibrin/HA-MA hydrogel formulations (Figure 3). No significant statistical differences between 4 mg/mL (Figure 3A) and 6 mg/mL (Figure 3B) fibrinogen groups were observed. However, the HA-MA concentration in each group influenced cellular activity. Within the two groups, there was significant statistical difference (p < 0.05) as calculated by one-way ANOVA. We, further, calculated the p values between individual group-pairs by student’s t-Test. With the introduction of HA-MA into the hydrogels, both 4 mg/mL and 6 mg/mL fibrinogen concentrations supported substantial cell activity with HA-MA concentrations below 1 mg/mL. Gel compaction occurred due to the stresses imposed by the BMSC contraction in the gel matrix [48]. Gel compaction could have been increased due to the presence of TGFβ [49,50]. Based on preliminary observations (data not shown), fibrinogen concentrations less than 3 mg/mL resulted in gel compaction after only two days in culture, after which cells migrated from inside the gel to the bare plate. This suggests that the hydrogels with fibrinogen concentrations below 3 mg/mL may be mechanically unreliable. 6 mg/mL was selected as the upper limit due to the fact that higher concentrations became too viscous to easily mix with the HA-MA solution. The BMSC viability was also visualized by live/dead staining (Figure 4), which showed increasing numbers of viable cells and cell density at successive time points for both 4 mg/mL and 6 mg/mL fibrinogen concentrations. It was evident that there were a greater number of cells outlined in day 6 compared to day 2 in both the 4 mg/mL and 6 mg/mL conditions. The use of both the metabolic assay and live/dead staining is a strong indication of cellular proliferation. Metabolic activity alone is not reliable enough to ensure cell division; however, the quantitative measure of cellular activity with the increase in cell density as determined by live/dead staining strongly suggests cellular proliferation. A decrease in cellular proliferation as a function of mechanical strength has been recently shown in similar hydrogels. Fibrin constructs containing HA modified with tyramine capable of crosslinking in the presence of hydrogen peroxide revealed a decrease in proliferation of endothelial cells with increased mechanical strength [51].

### Mechanical and structural characterization of fibrin/HA-MA hydrogels

**Figure 5:**
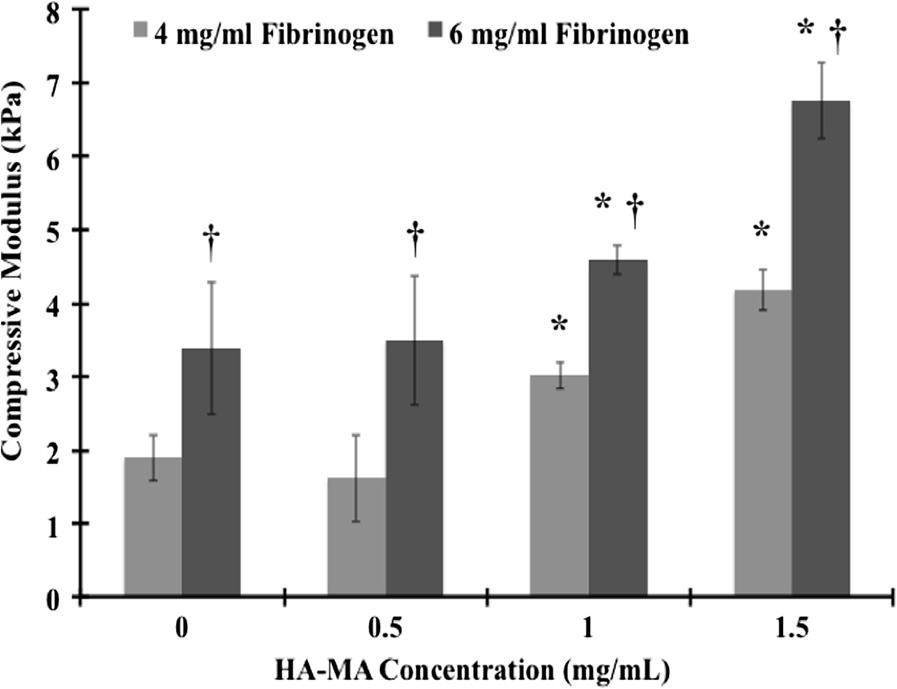
**Compressive modulus of fibrin/HA-MA hydrogels at 20% strain.** *Statistically significant difference between successive HA-MA concentrations for a given fibrinogen concentration (p < 0.05). † – Statistically significant difference between different fibrinogen concentrations for a given HA-MA concentration (p < 0.05).

To examine mechanical stiffness of the fibrin/HA-MA hydrogels, unconfined compression tests were performed to determine the compressive modulus at 20% strain (Figure 5), as described previously [47]. One-way ANOVA comparison of the data confirmed that the groups were statistically different (p < 0.05). Student’s t-Test was used to find the p value between two groups to determine any statistically significant difference (p < 0.05). Allowing HA-MA concentrations to vary from 0 to 1.5 mg/mL, the compressive modulus of the fibrin/HA-MA hydrogel with 4 mg/mL fibrinogen varied from 1.62 ± 0.6kPa to 4.19 ± 0.28kPa, and the compressive modulus of the fibrin/HA-MA hydrogel with 6 mg/mL fibrinogen varied from 3.39 ± 0.91kPa to 6.76 ± 0.52kPa. Since there was no statistically significant difference between the two conditions 4 mg/mL of fibrinogen with 0 mg/mL of HA-MA and 4 mg/mL of fibrinogen with 0.5 mg/mL of HA-MA (p = 0.1916), we decided to ignore the small apparent difference in the compressive moduli. The 6 mg/mL fibrinogen hydrogel possessed the higher compressive modulus at each HA-MA concentration. Increasing concentrations of both fibrinogen and HA-MA was directly correlated to an increased compressive modulus. Reinforcement with HA-MA crosslinking marginally improved mechanical strength over previously demonstrated pure fibrin hydrogels [42-45]. Since the gel was not stable below 3 mg/mL concentration, we could not measure the compressive strength at the lower concentrations.

**Figure 6:**
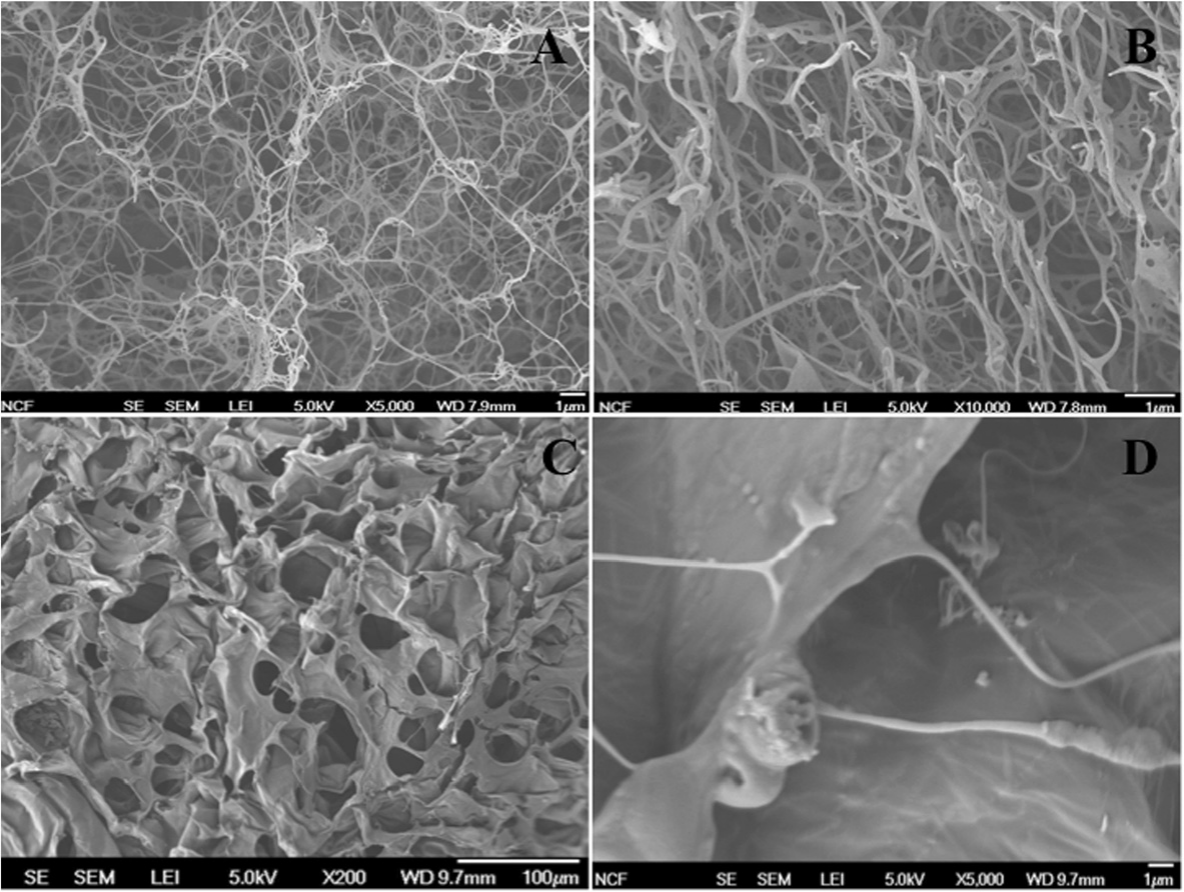
**Scanning electron micrographs of (A) 6 mg/mL fibrinogen with no HA-MA at 5000×, (B) 6 mg/mL fibrinogen with no HA-MA at 10,000×, (C) 6 mg/mL fibrinogen with 1 mg/mL HA-MA at 200x, and (D) 6 mg/mL fibrinogen with 1 mg/mL HA-MA at 5000×. **Scale bar: 1 μm.

Fibrinogen (fibrin-precursor) and HA-MA form a gel with an interpenetrating network of fibers through ionic and chemical interactions [34,51,52]. The hydrogel was then reinforced using UV photocrosslinking, which was achieved by photocrosslinking through the polymerizable vinyl group (–C = C–) present on the methacrylate side chain. The fibrin/HA-MA hydrogel even after crosslinking would still be completely biodegradable since the degradation of the hydrogel could be achieved through the hydrolysis of the ester bond present in the methacrylate side chain and the ether group present in the main hyaluronic acid backbone. To gain further insight into the structure of the hydrogels, field emission scanning electron microscopy (FESEM) was performed (Figure 6). The fibrin gels without HA-MA (Figures 6A and B) showed fibrous structure with pore sizes on the order of 1 μm. Addition of HA-MA resulted in a more sheet-like morphology with pore sizes in the range of 10-100 μm (Figure 6C). Magnifying further into the fibrin/HA-MA construct showed that fibrin fibers were still embedded within the structure (Figure 6D), indicating that the gelation occurred as intended by design: formation of fibrin gel followed by HA-MA reinforcement and crosslinking. Since 6 mg/mL fibrinogen with 1 mg/mL HA-MA led to well-controlled proliferation and an increase in mechanical strength, this fibrin/HA-MA hydrogel formulation was used for further study *in vitro* to determine mRNA expression of key chondrogenic markers.

### mRNA Expression analysis

**Figure 7:**
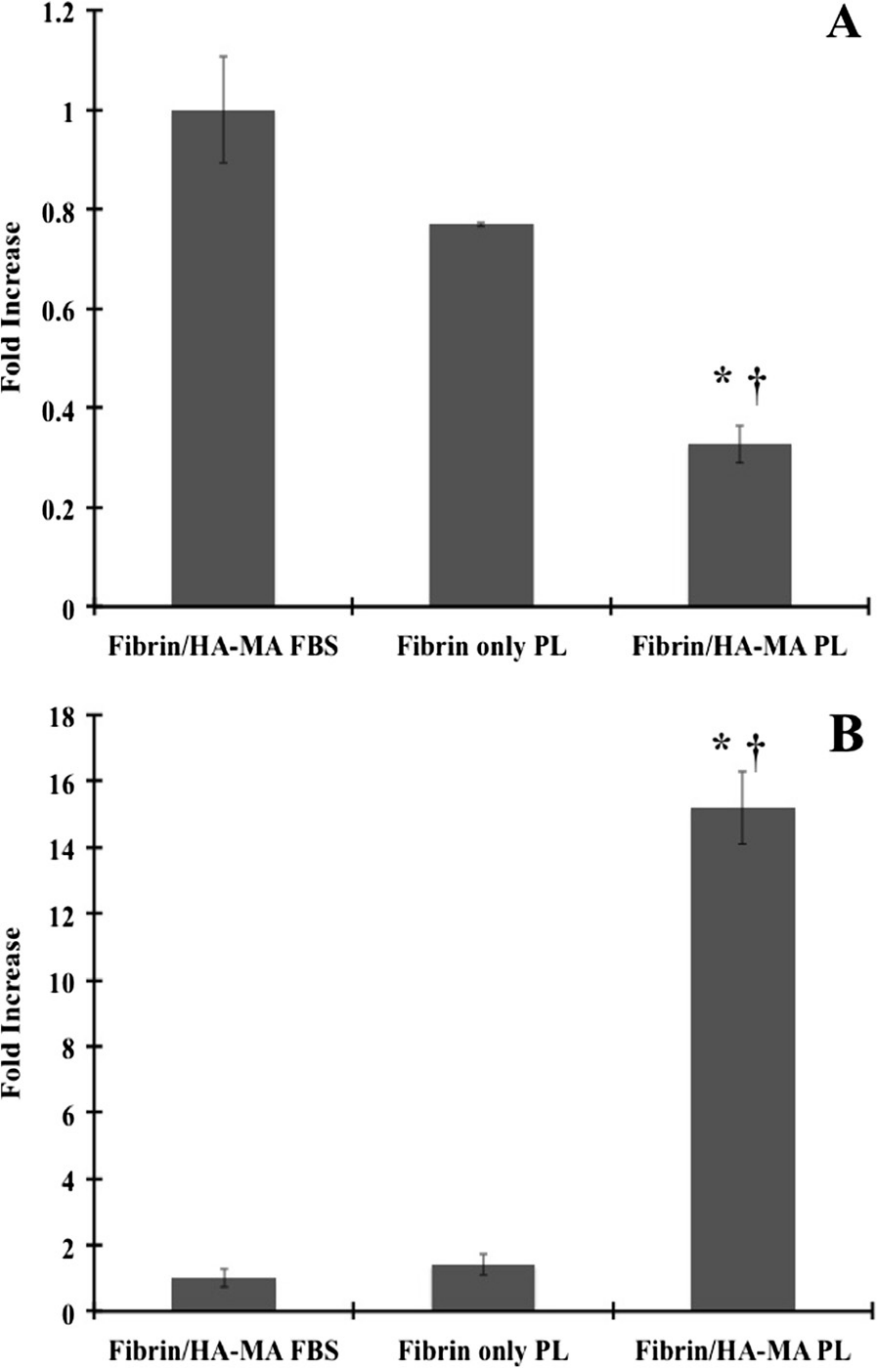
**mRNA expression for BMSCs. (A)** collagen type 1 alpha 1 gene and **(B)** SOX9 gene. All data has been normalized to fibrin/HA-MA with 10% FBS condition. Glyceraldehyde-3-Phosphate Dehydrogenase (GAPDH) is the housekeeping gene. * – Statistical significance from the fibrin/HA-MA with 10% FBS condition (p < 0.05) and † – Statistical significance from the Fibrin with PL condition (p < 0.05).

To determine chondrogenic potential, the cells were incubated within as-selected hydrogel for 12 days, and, the gene expression of collagen type 1 alpha 1 (COL1A1), collagen type 2 alpha 1 (COL2A1), SOX9 and aggrecan (ACAN) was assayed using qPCR (Figure 7). A platelet lysate (PL) was used as a supplementation to mimic real-life applications using BMSCs, since it is known to contain the growth factors such as transforming growth factor (TGFβ) that promote chondrogenesis [25]. While latent TGFβ was not quantified, active TGFβ concentration was quantified at 8.97 ng/mL using the Quantikine ELISA kit and other researchers have reported supplementation in the range of 5-20 ng/mL [53,54]. In the compared data set, one-way ANOVA confirmed that the data was statistically different (p < 0.05). The fold increase of mRNA transcripts was normalized to cells within fibrin/HA-MA gels supplemented with 10% fetal bovine serum (FBS). There was a downregulation of COL1A1 in all conditions with a significant downregulation in BMSCs in the fibrin/HA-MA containing PL (Figure 7A). SOX9 upregulation was observed for all conditions with a statistically significant difference in the fibrin/HA-MA with PL (Figure 7B). COL2A1 and ACAN genes were also upregulated in certain cases yet these genes were not expressed as strongly as SOX9 or COL1A1 (Refer to Additional file 1: Figure S1). A decrease in COL1A1 expression and an increase in SOX9 are good indicators of chondrogenesis [55]. Previous research has also indicated that the expressions of SOX9 and COL1A1 are very strong in comparison to the expression of COL2A1 and ACAN when BMSCs are cultured in a fibrin-based or hyaluronic acid-based scaffold [54,56-58]. Furthermore, SOX9 is by itself a favorable marker that indicates the production of COL2A1 and ACAN since it promotes the production of these extracellular components during chondrogenesis *in vivo*[59].

## Conclusion

The use of a composite hydrogel formulated with 6 mg/mL fibrinogen and 1 mg/mL HA-MA promoted optimal BMSC proliferation. Crosslinking reinforcement of the fibrin scaffold with HA-MA improved the compressive modulus of fibrin/HA-MA hydrogel. Increased expression of Sox9 and decreased expression of type I collagen demonstrated the chondrogenic potential of the fibrin/HA-MA composite hydrogel, and the fibrin/HA-MA system may indeed be a promising cell delivery vehicle capable of localizing BMSCs to the site of cartilage injury and promoting chondrogenesis.

## Materials and methods

### Materials

Dulbecco’s Modified Eagle Medium (DMEM), phosphate buffered saline (PBS), trypsin and alpha minimum essential media (AMEM) were obtained from Gibco Life Technologies (Grand Island, NY). Fetal bovine serum (FBS) was purchased from Atlas Biologicals (Fort Collins, CO), and doxycycline was purchased from APP Pharmaceutical (Schaumburg, IL). The antibodies CD105-Phycoerythrin (CD105-PE), CD73-Peridinin chlorophyll protein complex (CD73-PerCP), CD90-Allophycocyanin (CD-90APC), CD44-Fluorescein isothiocyanate (CD44-FITC) and CD34-FITC were purchased from BD Biosciences (Franklin Lakes, NJ). Bovine serum albumin (BSA), methacrylic anhydride (MA), hyaluronic acid (HA) sodium salt from Streptococcus equi, sodium hydroxide, fibrinogen from human plasma (50-70% protein, >80% clottable), aprotinin (3900-10400KIU/mg solid), Evithrom/Thrombin (800-1200 IU/mL) and ethanol (>99.5%) were obtained from Sigma Aldrich (St. Louis, MO). Dialysis membrane (MWCO 3500 Da) was purchased from VWR International (Denver, CO). For the cell live/dead assay, CellTrace™ Calcein Red-Orange, Sytox® Green and resazurin (PrestoBlue®) were purchased from Molecular Probes (Eugene, OR). The RealTime Ready Cell Lysis Kit, RNA Master Hydrolysis Probes kit, probes for aggrecan (ACAN), SOX9, collagen type 2 alpha 1 (COL2A1) and collagen type I alpha 1 (COL1A1) and left and right primers and the LightCycler II 480 polymerase chain reaction (PCR) platform were obtained from Integrated DNA Technologies (Coralville, IA). The Quantikine ELISA kit for quantifying TGFβ was obtained from R&D Systems (Minneapolis, MN).

### Equipment

Flow cytometry was performed on an Accuri C6 Flow Cytometer (BD Biosciences). Imaging for the live/dead assay was performed on a fluorescent microscope (AMG EVOS FL, Advanced Microscopy Group). To quantify cell growth, a Fluoroskan Ascent FL 2.5 fluorescent micro-plate reader with excitation/emission filters 544/590 (Thermo Scientific) was used. Hydrogel samples were uniaxially compressed on an Insight mechanical tensile system (MTS Systems). Proton nuclear magnetic resonance (^1^H NMR) spectroscopy was performed on a Varian INOVA 500 MHz spectrometer (International Equipment Trading Ltd.) with 5 mm triple resonance 1H/13C/15 N probe and sensitivity of 850:1. For field emission scanning electron microscopy (FESEM), the samples were sputter-coated with gold in a Cressington 108 Auto Sputter Coater (Cressington Scientific Instruments) and imaged under a JEOL JSM-7401 F field emission scanning electron microscope (JEOL USA Inc.). A LightCycler II 480 PCR platform (Integrated DNA Technologies) was used to analyze mRNA expression profiles.

### BMSC isolation and culture

Age, sex and number of donors are not disclosed since it is not a non-human subject research as determined by Colorado Multiple Institutional Review Board. This information was not available to us to ensure these donors cannot be traced. However, all donors undergoing a bone marrow nucleated cell procedure consented to providing bone marrow for research purposes. Bone marrow was fractionated by centrifugation and the buffy coat layer was isolated and plated at a cell seeding density of 1×10^6^ cells/cm^2^ in tissue culture flasks with DMEM supplemented with 10% FBS and 5 μg/mL doxycycline. Cells were incubated under 5% CO_2_ and 5% O_2_ at 37°C. Media was changed every two days, washing twice with PBS before adding fresh media. Between days 6 and 10, colony formation was assessed and cells were harvested using trypsin. Cells were counted and re-plated at 6000 cells/cm^2^ in AMEM with 10% FBS and 5 μg/mL doxycycline. Flasks were transferred to normoxic conditions with 21% O_2_ and 5% CO_2_ at 37°C. Media was changed every two days until cells were approximately 80% confluent.

### BMSC Verification

The human BMSC phenotype was verified in accordance with the Mesenchymal and Tissue Stem Cell Committee of the International Society for Cellular Therapy criteria: the surface protein expression must be positive for CD105, CD73 and CD90 and negative for at least one of the following: CD45, CD34, CD14 or CD11b [60]. CD44 has also been documented as a phenotypical MSC marker [61-63] and was also assayed for. CD105-PE, CD73-PerCP, CD90-APC, CD44-FITC and CD34-FITC surface markers were assessed using an Accuri C6 Flow Cytometer. Approximately 50,000 cells per surface protein, plus another 50,000 for four-color analysis, were centrifuged to a pellet and washed in PBS containing 2% BSA. The antibody surface markers (CD105-PE, CD73-PerCP, CD90-APC, CD44-FITC and CD34-FITC), at a concentration of 5 μg/mL, were added with cells and incubated in 2% BSA/PBS solution for 1 hour. Unbound antibody was removed; the cells were re-suspended in PBS to be run through the flow cytometer, gated at 10,000 events. An unlabeled population was run as a control to determine gating for the detection of positive and negative shifts in fluorescence; gating remained constant for all runs.

### Modification of HA

The modification of HA was performed as described previously [40]. Briefly, HA sodium salt from Streptococcus equi (MW 1.5-1.8 MDa) was suspended to a final 1% (w/v) solution in diH_2_O. The solution was placed in a round bottom flask and cooled to 5°C in a water bath with stirring. A 10 molar excess of MA was added to the flask and the solution was maintained at pH 10 using 5 N NaOH. After 24 hours, the reaction mixture was slowly added to excess ethanol for precipitation. The precipitate was centrifuged and the supernatant was decanted. The ethanol precipitation was repeated once more to further remove any unreacted MA. The HA-MA was transferred to a round bottom flask to vacuum dry on a rotary evaporator to remove excess ethanol. After drying, the sample was dissolved in diH_2_O and dialyzed for three days against diH_2_O with refreshment of water every day. The sample was then transferred to a 50 mL conical tube and lyophilized under vacuum. The MA conversion was determined using ^1^H NMR in D_2_O by the ratio of the integral values between the MA proton peaks at 5.6 ppm and 6.1 ppm to the methyl proton peak of HA at 1.9 ppm.

### Fibrin/HA-MA hydrogel preparation with BMSCs

For BMSC culture, fibrinogen (final concentration: 4 mg/mL or 6 mg/mL), aprotinin (30 KIU/mL), doxycycline (5 μg/mL) and HA-MA (0.5, 1.0 or 1.5 mg/mL) were mixed thoroughly in 200 μL AMEM with 1% (w/v) photoinitiator (Irgacure 2959) and BMSCs (10,000 cells/well) in 96-well plate. To form fibrin gel, thrombin was added at 1 U/mL. Upon complete gelation, the gels were exposed to UV light (10 mW/cm^2^) at, approximately, 350 nm for 5 minutes to crosslink HA-MA. The samples were limited to a 5-minute exposure to minimize the negative effects of UV on the hydrogel. BMSCs were cultured at 37°C under 5% CO_2_ using AMEM with 10% FBS and 5 μg/mL doxycycline. Exposure to UV light was limited to 5 minutes since it could have a negative effect on proteins and DNA within the cells.

### BMSC viability and proliferation

BMSC viability and proliferation within fibrin/HA-MA gels was investigated using live/dead staining and metabolic activity assay at 2, 4 and 7 days. CellTrace™ Calcein Red-Orange was added to a final concentration of 2 μM, with respect to the total volume of gel and media, and incubated for 15 minutes prior to observation. Similarly, Sytox® Green was added to a final concentration of 2.5 μM and incubated for 5 minutes. After incubation, BMSC viability was observed by fluorescence microscopy. To quantify cell growth, cultures were exposed to PrestoBlue®. A 10-fold dilution of PrestoBlue® with respect to the total gel and media volume was added to the media and incubated for 2 hours on an orbital shaker. The media was removed and transferred to a 96-well plate, and fluorescence was measured by microplate reader.

### Mechanical testing

Hydrogels were prepared in 24-well plate to a final volume of 1 mL as described above without the addition of BMSCs. PBS was added to allow the final gels to be fully hydrated for 24 hours. Unconfined compression testing with a 5 N load cell was performed using a 5kN MTS Insight load frame material testing system. Compression testing was performed at a 2 mm/min strain rate to 80% strain. A stress-strain curve was plotted and an exponential regression model was fitted to the data. The modulus was calculated in the initial linear region at 20% strain.

### Field emission scanning electron microscopy

Hydrogels were prepared as described above to a final volume of 1 mL in 3 mL syringes with the tip removed. Fibrin-only gels with a final concentration of 6 mg/mL fibrinogen were compared to the same gels containing 1 mg/mL HA-MA. The samples were lyophilized until dry and then cross-sectioned. The samples were placed in a vacuum chamber to sputter-coat a thin layer of gold evenly across the sample surface for 40s at an operating current of 40 mA. The sample was then loaded into the field emission scanning electron microscope for morphological imaging. The sample was imaged under an operating voltage of 5 kV.

### mRNA expression analysis

To determine potential chondrogenesis, BMSCs were cultured in 6 mg/mL fibrinogen gels with/without 1 mg/mL HA-MA for 12 days with the supplementation of 10% Platelet lysate (PL) obtained using osmotic stress from the platelet rich fraction of human whole blood. PL was prepared from the same blood draw and donor as to eliminate discrepancies in growth factor concentrations between conditions. Gels were harvested and washed with PBS, centrifuged to a pellet and the supernatant decanted. The probes for aggrecan (ACAN), SOX9, collagen type 2 alpha 1 (COL2A1) and collagen type 1 alpha 1 (COL1A1), and left and right primers were mixed according to manufacturer’s instructions with template RNA from the samples. Solutions were added to each well of a 96-well plate and transferred to the LightCycler II 480 PCR platform. A one-step reaction protocol was programmed into the machine according to the manufacturer’s instructions. RealTime ready Cell Lysis Kit contained a cell lysis solution and RNase inhibitor that was added to each gel in volumes of 39.5 μL and 0.5 μL, respectively. This was incubated on ice for 5 minutes and mixed thoroughly to extract mRNA from cells within the gel. Two master mixes were prepared before lysis, one containing primers and probes and the other containing Tth deoxyribonucleic acid (DNA) polymerase (capable of RNA transcription), deoxyribonucleotide triphosphates (dNTPs), an activator and an enhancer. The primers and probes were mixed to final concentrations of 5 μM probe and 5 μM total left and right primer. Template, PCR grade water and both master mixes were combined in ratios based on the manufacturer’s instructions yielding a final primer and probe concentration of 0.325 μM. Final reaction volume was 20 μL/well run in triplicates for each probed mRNA. Glyceraldehyde-3-Phosphate Dehydrogenase (GAPDH) was used as a housekeeping gene with primers and probes provided within the qPCR kit. The relative expression of each mRNA was determined by comparing the critical threshold values of the target genes, i.e. SOX9, COL2A1, COL1A1 & ACAN, to that of the housekeeping gene, GAPDH. Table 1 indicates the sequences of primers for the various target genes. Fold expression of each condition was determined by normalizing the relative expression of the target sample with a reference sample of BMSCs cultured in fibrin/HA-MA hydrogels substituted with 10% FBS. Active TGFβ concentration was determined using the Quantikine ELISA Kit.

**Table 1:** Gene sequences for mRNA expression during chondrogenesis

**Chondrogenic marker**	**Symbols**	**mRNA Sequence**	
		**Left primer**	**Right primer**
Glyceraldehyde-3-Phosphate Dehydrogenase^†^	GAPDH	tagtagccgggccctacttt	tcctcctgtttcatccaagc
Aggrecan	ACAN	cctccccttcacgtgtaaaa	gctccgcttctgtagtctgc
Collagen Type 1 Alpha 1	COL1A1	gggattccctggacctaaag	ggaacacctcgctctcca
Collagen Type 2 Alpha 1	COL2A1	agggccaggatgtccatt	aggagagggcccacagag
SRY-box containing gene 9	SOX9	gtacccgcacttgcacaac	tctcgctctcgttcagaagtc

^†^ - Glyceraldehyde-3-Phosphate Dehydrogenase (GAPDH) is the housekeeping gene.

### Statistical analysis

All experiments were conducted with a sample size of five unless otherwise noted with values reported as the mean ± the standard deviation. One-way ANOVA was used to calculate p-values to determine over all statistical significance between all groups (p < 0.05). Unpaired Student’s t-Tests were used to calculate p-values to determine statistical significance (p < 0.05).

## Abbreviations

AC: Autologous chondrocytes; BMSC: Bone marrow-derived mesenchymal stem cells; HA: Hyaluronic Acid; HA-MA: Hyaluronic Acid Methacrylate; MSC: Mesenchymal stem cells; OA: Osteoarthritis; PE: Phycoerythrin; PerCP: Peridinin chlorophyll protein complex; APC: Allophycocyanin; FITC: Fluorescein isothiocyanate; BSA: Bovine serum albumin; PBS: Phosphate buffered saline; PL: Platelet lysate; SSC: Side scatter channel; FSC: Forward scatter channel; FL: Fluorescent channel; mRNA: Messenger Ribonucleic Acid; qPCR: Quantitative Polymerase Chain Reaction; TGFβ: Transforming growth factor *beta* .

## Competing interests

The authors declare that they have no competing interests.

## Authors’ contributions

TS carried out all experiments, contributed to experimental designs and protocols and drafted the manuscript. KM helped with data analysis, writing and finalizing manuscripts. MI provided guidance on discussion and helped with finalizing the manuscripts. RD provided guidance with experimental designs and protocols and provided conceptual input and troubleshooting. DP oversaw the project providing conceptual development, advice in experimental designs and protocols, troubleshooting and finalizing the manuscript. All authors read and approved the final manuscript.

## Supplementary Material

Additional file 1: Figure S1 mRNA expression for BMSCs. (A) aggrecan gene and (B) collagen type 2 alpha 1 gene. All data has been normalized to fibrin/HA-MA with 10% FBS condition. Glyceraldehyde-3-Phosphate Dehydrogenase (GAPDH) is the housekeeping gene. * – Statistical significance from the Fibrin with 10% FBS condition (p < 0.05). Click here for file
