# Erratum to: A fibrin/hyaluronic acid hydrogel for the delivery of mesenchymal stem cells and potential for articular cartilage repair

**DOI:** 10.1186/1754-1611-8-27

**Published:** 2014-11-28

**Authors:** Timothy N Snyder, Krishna Madhavan, Miranda Intrator, Ryan C Dregalla, Daewon Park

**Affiliations:** Bioengineering Department, University of Colorado, Anschutz Medical Campus, Mail Stop 8607, 12700 East 19th Avenue, Aurora, CO 80045 USA; Regenerative Sciences, 403 Summit Blvd, Suite 201, Broomfield, CO 80021 USA

The authors would like to add the following detail to the Competing Interests and Acknowledgments stated in the published article [1] “At the time of writing, TNS and RCD were employees of Regenerative Sciences, LLC. The authors also would like to especially thank Dr. Christopher Centeno and Regenerative Sciences for partially supporting and funding this project.”

## References

[1] Snyder TN, Madhavan K, Intrator M, Dregalla RC, Park D (2014). A fibrin/hyaluronic acid hydrogel for the delivery of mesenchymal stem cells and potential for articular cartilage repair. J Biol Eng.

