# Supplementary figures and images for: A fibrin/hyaluronic acid hydrogel for the delivery of mesenchymal stem cells and potential for articular cartilage repair
Source: J Biol Eng. 2014 May 1;8:10. doi: 10.1186/1754-1611-8-10 (PMC4109069; doi:10.1186/1754-1611-8-10)

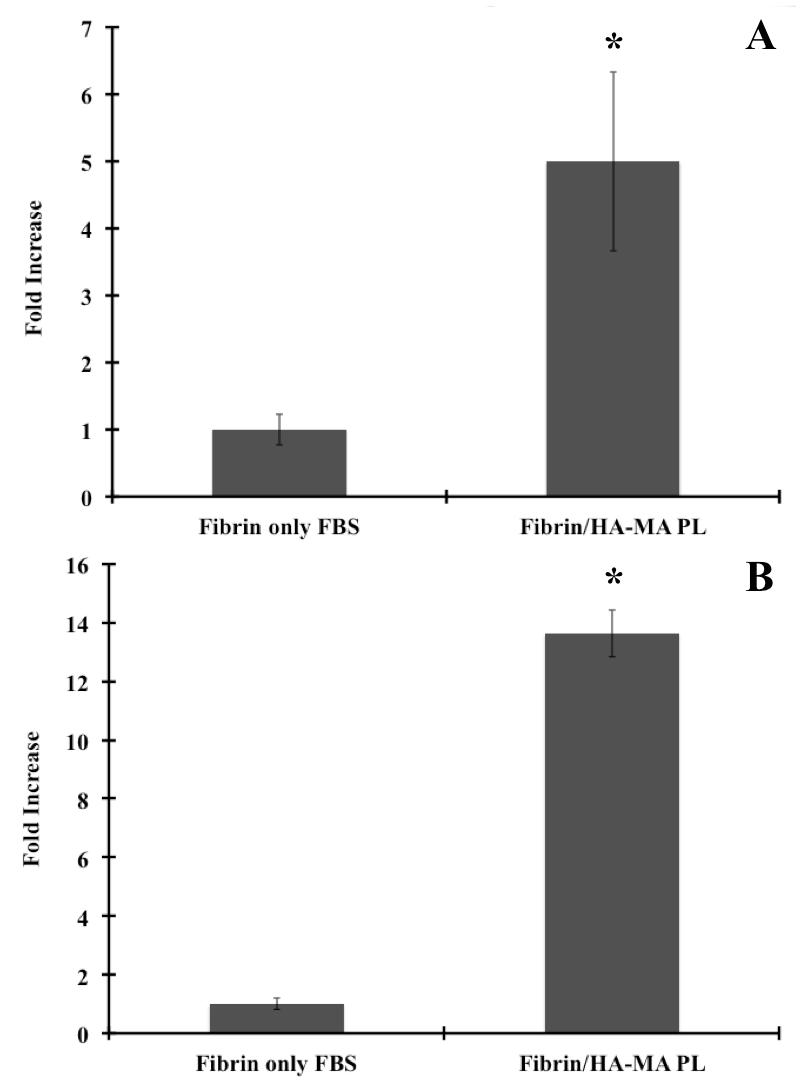

Supplement: Additional file 1: Figure S1 — mRNA expression for BMSCs. (A) aggrecan gene and (B) collagen type 2 alpha 1 gene. All data has been normalized to fibrin/HA-MA with 10% FBS condition. Glyceraldehyde-3-Phosphate Dehydrogenase (GAPDH) is the housekeeping gene. * – Statistical significance from the Fibrin with 10% FBS condition (p < 0.05). [file 1754-1611-8-10-S1.bmp]
